# Immunodeficiency, centromeric instability, and facial anomalies (ICF) syndrome identified by whole-exome sequencing (WES): a case report from a developing country

**DOI:** 10.1093/omcr/omaf079

**Published:** 2025-06-27

**Authors:** Rahaf Joma, Shahed Radwan, Sakhaa Hannoun, Jawad Hasson, Banan M Aiesh

**Affiliations:** Department of Medicine, College of Medicine and Health Sciences, An-Najah National University, Nablus 44839, Palestine; Department of Medicine, College of Medicine and Health Sciences, An-Najah National University, Nablus 44839, Palestine; Department of Pediatric Medicine, PGY2, Pediatric Residency Program, Rafidia Surgical Hospital, Nablus 44839, Palestine; Department of Pediatric Medicine, Rafidia Surgical Hospital, Nablus 44839, Palestine; Department of Infection Prevention and Control, An-Najah National University Hospital, Nablus 44839, Palestine

**Keywords:** ICF syndrome, centromeric instability, facial dysmorphism syndrome, immunoglobulins

## Abstract

Background: Immunodeficiency, centromeric instability, and facial anomalies (ICF) syndrome is a rare genetic autosomal recessive disorder that results in impaired immune system function, instability of the centromeric region of chromosomes, and distinct facial features. This is the first case report of ICF in Palestine. Case presentation: A male child with recurrent respiratory tract infections, ear discharge and facial anomalies. Whole-exome sequencing was performed. A homozygous missense variant DNMT3B was identified, and the patient was diagnosed with ICF and was managed successfully with intravenous immunoglobulins. Conclusion: ICF syndrome is a rare genetic disorder that affects the immune system. It has three common symptoms which are present in most cases. Treatments like immunoglobulin supplementation or allogeneic stem cell transplantation can improve the chances of survival and enhance the quality of life.

## Introduction

Immunodeficiency, centromeric instability, and facial anomalies (ICF) is an autosomal recessive disease characterized by immunodeficiency, centromeric instability, and facial anomalies. In 1978, it was described in patients with a variable primary immunodeficiency disease (PID) and centromere instability [1]. In the first year of life, patients with ICF syndrome usually require hospitalization due to severe recurrent respiratory tract and gastrointestinal infections. There are also growth delays, failures to thrive, psychomotor impairments, and mild facial dysmorphisms [2].

ICF has been described in about 118 patients worldwide [3]. Chromosome breaks were observed in these patients due to distinctive rearrangements along the centromeres (the juxtacentromeric heterochromatin) of chromosomes 1 and 16, and occasionally 9 [1]. ICF type 1 accounts for approximately 50% of ICF patients who had mutations in the DNMT3B gene [2]. The remaining half may be caused by mutations in ZBTB24 (ICF2), CDCA7 (ICF3), or HELLS (ICF4).

Here, we report a novel homozygous mutation in DNMT3B gene in a Palestinian male child with ICF type 1. It is the first reported ICF case in Palestine. Whole-exome sequencing (WES) reported that the patient’s variant was not described before, and it lies within the catalytic domain.

DNMT3B is the major de novo DNA methyltransferase expressed and active during the early stages of embryonic development. It is located on chromosome 20 at position 20q11.2 [4]. Recent observations suggest that DNMT3B acts as the main enzyme methylating intragenic regions of active genes [5]. However, complete loss of function of this gene leads to embryonic mortality in mice. Studies on murine models suggest that DNMT3B mutations do not affect the development of normal T cells at birth but modify their survival in the thymus by promoting their death through a p53-independent mechanism. Altered DNA methylation patterns contribute to immunological dysregulation and the pathogenesis of systemic autoimmune disorders by controlling gene expression. in the Middle East, few number of cases has been reported (11 cases); five in Saudi Arabia [6], four in Lebanon [7], and two in Iran [4]. In conclusion, releasing this first case report in Palestine is critical for expanding global awareness of ICF syndrome, demonstrating the utility of WES in uncommon genetic illnesses, and encouraging additional research that can benefit in the diagnosis and treatment of comparable patients worldwide.

## Case report

A male child who is now 8 years old was a product of normal vaginal delivery who was born at term with a birth weight of 2.6 kg. After delivery, He was admitted to the neonatal intensive care unit (NICU) for one week owing to feeding difficulties caused by cleft lip and palate. His parents are first cousins with no history of genetic diseases. He has two healthy siblings, and one younger sister with the same condition. At the age of two months, he had frequent symptoms of high-grade fever, diarrhea, upper respiratory tract infection symptoms, and ear discharge, with minimal improvement between each episode of illness. At six months age, he received vaccinations for DPT, Hib, Hepatitis B, and OPV3 according to the Palestinian vaccination schedule. Unfortunately, he had high-grade fever and was hospitalized. His condition worsened, leading to cyanosis and desaturation, requiring transfer to the pediatric intensive care unit (PICU). Due to aspiration pneumonia, he was mechanically ventilated for one month necessitating WES screening.

**Figure 1:**
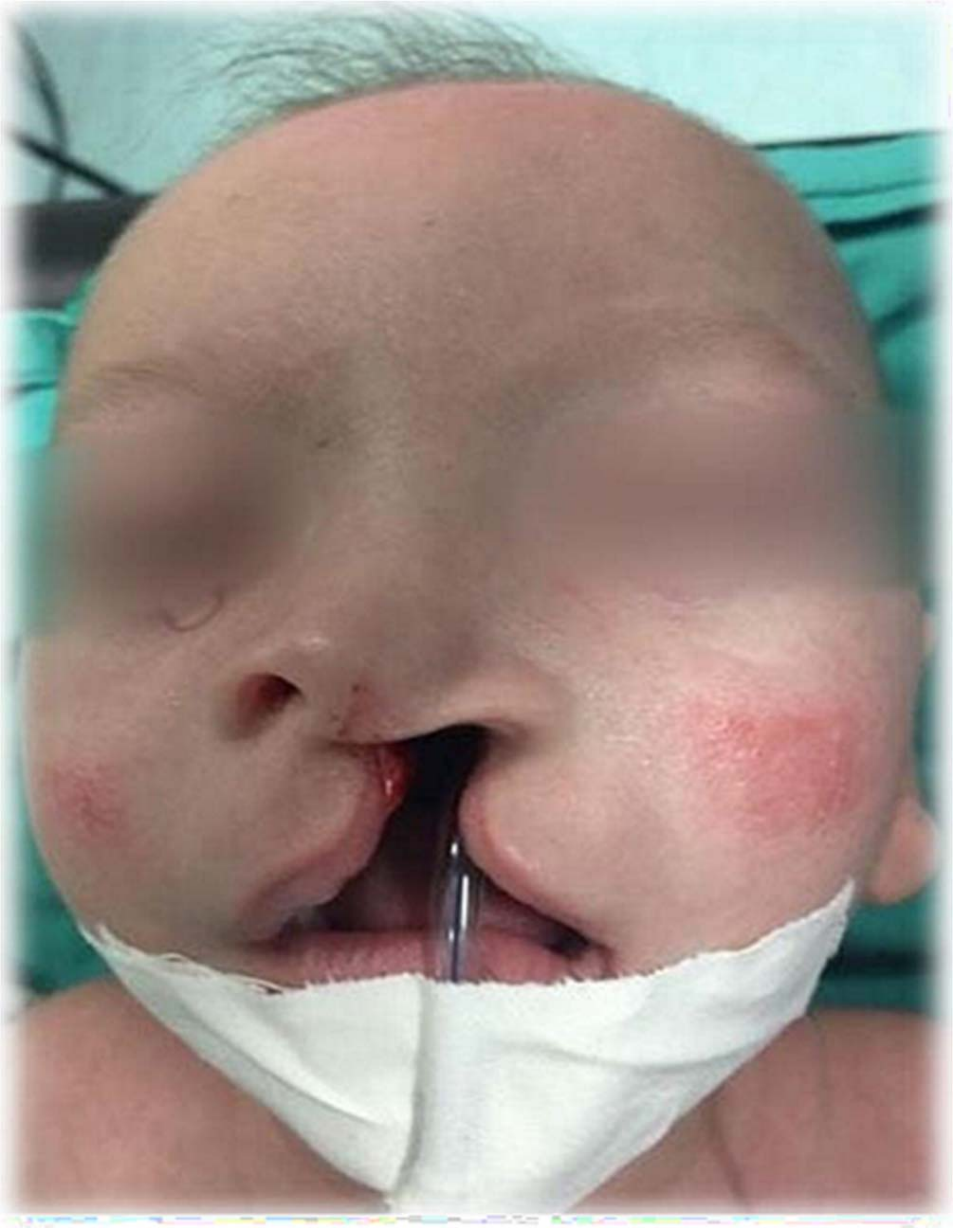
Facial anomalies (cleft lip and palate and flat nasal bridge) for the child at the age of 4 months.

Regarding growth parameters, including weight, height, and head circumference, they were below the 3^rd^ percentile. Additionally, he had distinct dysmorphic features such as cleft lip and palate and flat nasal bridge (Fig. 1). Developmental milestones were within average, apart from language delay due to cleft palate and facial anomalies. Full investigations revealed microcytic anemia with low iron study, high thyroid-stimulating hormone (TSH), low thyroxine (T4), peak growth hormone, normal adrenocorticotropic hormone (ACTH), with normal renal and liver function tests. Serum immunoglobulin analysis showed hypogammaglobinemia (low IgM, IgA, and IgG). A bone age study was conducted and showed delayed bone age. Brain magnetic resonance imaging and echocardiogram showed no abnormalities. For genetic testing, WES was performed using Agilent V6 + UTR library preparation and the Illumina NextSeq 500 sequencing platform. A homozygous missense variant DNMT3B (LRG_56t1: C.2476C>T; p. Arg826Cys) was identified, and the patient was diagnosed with type 1 ICF syndrome.

For management, the patient started receiving intravenous immunoglobulin (IVIG) of 10 g every 4 weeks, aiming to keep his IgG level above 600 mg/dl (Table 1). Additionally, he has maintained on daily oral l-thyroxine 25 mcg, oral iron drops, and growth hormone 0.4 mg daily. The cleft lip was repaired at the age of 4 months, and the cleft palate was fixed at the age of 8 months. Currently, he receives IVIG every 6 to 10 weeks. The IgG level improved significantly (between 550 and 600 mg/dl). Follow-up TSH, T4, and triiodothyronine (T3) levels were acceptable. However, there was no improvement in the iron study or hemoglobin level and was followed up with a hematologist for intravenous iron. He continues to have diarrhea, stomatitis, and upper respiratory tract symptoms, which are managed at home with nebulizers. Due to experiencing fever and pneumonia, he has to be admitted to the hospital once or twice a year.

**Table 1:** Serum immunoglobulin levels in response to intravenous immunoglobulin administration.

**Immunoglobulins type**	**Immunoglobulin levels before each IVIG dose (mg/dl)**	**Immunoglobulin reference range (mg/dl)**	**Age**
IgA	0	8.1–68	6 months
IgG	8	215–704	
IgM	14	35–102	
IgA	2697	14–106	12 months
IgG	29	345–1213	
IgM		43–173	
IgA	9	33–202	6 years
IgG	551	633–1280	
IgM	0	48–207	
IgA	2	33–202	7 years
IgG	612	633–1280	
IgM	1	48–207	

## Discussion

ICF is an autosomal recessive disease characterized by immunodeficiency, centromeric instability, and facial anomalies. ICF patients experience opportunistic infections such as bronchopneumonia and otitis, suffer from diarrhea, have low birth weight, and may experience cognitive impairment and developmental delays. This is mainly caused by a homozygous DNMT3B mutation [2]. Maraschio later named the illness ICF, using the diagnostic triad of facial dysmorphism, chromosomal abnormalities, and variable immunodeficiency. There are four categories of ICF, and ICF type 1 (DNMT3B) is the most common, accounting for approximately 50% of all cases in which affected cases often lose their lives in the first or second decade of life [2]. Types 2, 3, and 4 are characterized by mutations in the ZBTB24, CDCA7, and HELLS genes, respectively. Eleven cases of ICF syndrome have been reported in the Middle East: five in Saudi Arabia [6], four in Lebanon [7], and two in Iran [4]. No cases have been reported in Palestine before.

Our case, who was later on diagnosed with type 1 ICF, had a history of NICU admission for one week due to feeding issues resulting from a cleft lip and palate. His growth parameters were below the 3^rd^ percentile and he has been experiencing frequent symptoms of high-grade fever, diarrhea, upper respiratory tract infections, and ear pain since early infancy. Meningitis, bilateral lung infiltration and pleural effusion, fever, and otitis media were reported in the presentation of Saudi cases [6]. The disease phenotype is characterized by recurring severe pulmonary or gastrointestinal infections in early childhood and micrognathia [3].

At the age of six months, after receiving his vaccinations, he was admitted to the pediatric ward due to high-grade fever and was intubated on a mechanical ventilator for one month in the PICU due to aspiration pneumonia necessitating WES screening. Other cases were screened and diagnosed with ICF following presentation with pneumonia [4], or from an ear infection and neck abscess. WES reported that this patient’s variant was not described before, and lies within the catalytic domain, leading to the diagnosis of ICF type 1 case with DNMT3B mutation. A similar variant (p.Arg840Gln in the older annotation used OMIM 6029000012) was previously reported in a Japanese ICF patient at part of a compound heterozygous mutation [8]. Patients with the ICF may have lower levels of immunoglobulins, such as IgG, IgG subclasses, IgA, and/or IgM; as presented in this case and Irani case [4].

Patients diagnosed with ICF syndrome can benefit significantly from IVIG therapy or allogeneic stem cell transplantation [9]. Our patient received IVIG with a significant improvement in his IgG levels, which has been maintained between 550 and 600 mg/dL. This underscores the importance of tailoring treatment approaches based on the clinical manifestations of the disease. It is also important to note that live-attenuated vaccines should be strictly avoided in such cases because of their immunodeficiency. Moreover, this case report highlights the significance of early screening and diagnosis for patients born into families with a history of primary immunodeficiency.

## Supplementary Material

ICF_CARE_omaf079
