# Supplementary material for: Immunodeficiency, centromeric instability, and facial anomalies (ICF) syndrome identified by whole-exome sequencing (WES): a case report from a developing country
Source: Oxf Med Case Reports. 2025 Jun 27;2025(6):omaf079. doi: 10.1093/omcr/omaf079 (PMC12202303; doi:10.1093/omcr/omaf079)
Supplement: ICF_CARE_omaf079 [file icf_care_omaf079.pdf]

CARE case report guidelines

CARE Checklist of information to include when writing a case report

(cc) BY-NC-ND

Topic	Item	Checklist item description	Reported on Line
Title	1	The diagnosis or intervention of primary focus followed by the words "case report"	P1. L1 and L2
Key Words	2	2 to 5 key words that identify diagnoses or interventions in this case report, including "case report"	P2 . L63 and L64
Abstract (no references)	3a	Introduction: What is unique about this case and what does it add to the scientific literature?	P2 .L38_47
	3b	Main symptoms and/or important clinical findings	P2 . L49_50
	3c	The main diagnoses, therapeutic interventions, and outcomes	Not applicable
	3d	Conclusion—What is the main "take-away" lesson(s) from this case?	P2 . L57_61
Introduction	4	One or two paragraphs summarizing why this case is unique (**may include references**)	P3. L70_87
Patient Information	5a	De-identified patient specific information.	P4. L91_93
	5b	Primary concerns and symptoms of the patient.	P4. L 98_102
	5c	Medical, family, and psycho-social history including relevant genetic information	P4. L 94_102
	5d	Relevant past interventions with outcomes	P5. L 128_131
Clinical Findings	6	Describe significant physical examination (PE) and important clinical findings.	P4 .L 103_112
Timeline	7	Historical and current information from this episode of care organized as a timeline	Not applicable
Diagnostic Assessment	8a	Diagnostic testing (such as PE, laboratory testing, imaging, surveys).	P4-5 .L 112_115
	8b	Diagnostic challenges (such as access to testing, financial, or cultural)	Not applicable
	8c	Diagnosis (including other diagnoses considered)	P5 .L 114_115
	8d	Prognosis (such as staging in oncology) where applicable	Not applicable
Therapeutic Intervention	9a	Types of therapeutic intervention (such as pharmacologic, surgical, preventive, self-care)	P6. L148_154
	9b	Administration of therapeutic intervention (such as dosage, strength, duration)	P6. L148_154
	9c	Changes in therapeutic intervention (with rationale)	Not applicable
Follow-up and Outcomes	10a	Clinician and patient-assessed outcomes (if available)	Not applicable
	10b	Important follow-up diagnostic and other test results	Not applicable
	10c	Intervention adherence and tolerability (How was this assessed?)	Not applicable
	10d	Adverse and unanticipated events	Not applicable
Discussion	11a	A scientific discussion of the strengths AND limitations associated with this case report	P7 .L 170, P 8. L205_207
	11b	Discussion of the relevant medical literature **with references**.	P6_9 . L 156_ 221
	11c	The scientific rationale for any conclusions (including assessment of possible causes)	P9. L210_216
	11d	The primary "take-away" lessons of this case report (without references) in a one paragraph conclusion	P9 .L 222_225
Patient Perspective	12	The patient should share their perspective in one to two paragraphs on the treatment(s) they received	Not applicable
Informed Consent	13	Did the patient give informed consent? Please provide if requested	Yes ☑ No ☐
